# SARS-CoV-2 infection in IVF-conceived early pregnancy and the risk of miscarriage: a matched retrospective cohort study

**DOI:** 10.1093/hropen/hoae024

**Published:** 2024-04-23

**Authors:** Jian Xu, Di Mao, Chunlin Liu, Ling Sun

**Affiliations:** Department of Obstetrics and Gynaecology, Center of Reproductive Medicine, Guangzhou Women and Children’s Medical Center, Guangzhou Medical University, Guangzhou, China; Department of Obstetrics and Gynaecology, Center of Reproductive Medicine, Guangzhou Women and Children’s Medical Center, Guangzhou Medical University, Guangzhou, China; Department of Obstetrics and Gynaecology, Center of Reproductive Medicine, Guangzhou Women and Children’s Medical Center, Guangzhou Medical University, Guangzhou, China; Department of Obstetrics and Gynaecology, Center of Reproductive Medicine, Guangzhou Women and Children’s Medical Center, Guangzhou Medical University, Guangzhou, China

**Keywords:** SARS-CoV-2, infection, IVF, early pregnancy, miscarriage

## Abstract

**STUDY QUESTION:**

Is SARS-CoV-2 infection in IVF-conceived early pregnancy associated with a higher risk of miscarriage?

**SUMMARY ANSWER:**

Infection with SARS-CoV-2 during early pregnancy in women conceiving by IVF may not be associated with an increased rate of miscarriage.

**WHAT IS KNOWN ALREADY:**

In naturally conceived pregnancies, most findings have shown that SARS-CoV-2 infection does not increase the risk of miscarriage, while some studies have shown that SARS-CoV-2 infection is associated with a higher risk of miscarriage.

**STUDY DESIGN, SIZE, DURATION:**

A matched retrospective cohort study was conducted in a tertiary hospital-based reproductive medicine center. The infection group included women who contracted coronavirus disease 2019 (COVID-19) before 20 weeks gestation from 6 December 2022 to 10 January 2023. Each infected woman was matched with three historical control subjects from 1 January 2018 to 31 May 2022.

**PARTICIPANTS/MATERIALS, SETTING, METHODS:**

The infection group was matched with historical control subjects based on female age (±1 year), number of gestational sacs, number of previous miscarriages, BMI (±2 kg/cm^2^), main causes of infertility, gestational week, and fresh versus frozen embryo transfer.

**MAIN RESULTS AND THE ROLE OF CHANCE:**

A total of 150 pregnant women infected with COVID-19 before 20 weeks of gestation were included in the infection group, which was matched at a 3:1 ratio with 450 historically pregnant controls. There were no significant differences in age, BMI, and endometrial thickness between the two groups. The overall incidence of miscarriage was not significantly different between the infection group and the control group (4.7% versus 5.8%, *P* = 0.68). When the infection group was stratified into three subgroups based on the gestational age at the onset of infection (0–7 + 6, 8–11 + 6, and 12-19 + 6 weeks), no significant differences were observed in the incidence of miscarriage between the infection group and the matched control group in any of the subgroups (9.8% versus 13.8%, *P* = 0.60; 5.4% versus 4.5%, *P* = 1.00; and 1.4% versus 1.9%, *P* = 1.00, respectively).

**LIMITATIONS, REASONS FOR CAUTION:**

The major limitation of this study is the relatively small sample size; therefore, caution is suggested when drawing any definitive conclusions. Nonetheless, our study is the largest sample study of the influence of COVID-19 infection on the miscarriage rate in early pregnancy after IVF.

**WIDER IMPLICATIONS OF THE FINDINGS:**

Our findings may provide important insights for reproductive physicians and obstetricians during preconception and early pregnancy counseling.

**STUDY FUNDING/COMPETING INTEREST(S):**

This study was supported by the Natural Science Foundation of Guangdong Province (No. 2023A1515010250). The authors report no conflicts of interest.

**TRIAL REGISTRATION NUMBER:**

N/A.

WHAT DOES THIS MEAN FOR PATIENTS? Since the start of the coronavirus disease 2019 (COVID-19) pandemic in early 2020, many women around the world have been infected with SARS-CoV-2 during the early stages of pregnancy. These women have wanted to know whether the infection increases the risk of miscarriage. However, this question remains unclear, especially in pregnant women after IVF and embryo transfer.

To address this gap in the field of IVF, we conducted a matched retrospective cohort study involving 150 pregnant women infected with COVID-19 and 450 historically pregnant controls. Our findings suggest that women conceiving by IVF and infected with SARS-CoV-2 during early pregnancy may not face an increased rate of miscarriage. For women who have experienced both COVID-19 infection and miscarriage during early pregnancy, our results provide meaningful clinical evidence that the COVID-19 infection may not be the causative factor for the miscarriage.

## Introduction

Since the start of the coronavirus disease 2019 (COVID-19) pandemic in early 2020, whether COVID-19 infection in early pregnancy increases the risk of miscarriage is a source of serious concern for both pregnant women and obstetricians. However, this question remains controversial since the studies to date have drawn mixed conclusions.

In the early stages of the pandemic, two small-sample studies found no correlation between COVID-19 infection and miscarriage (Cosma *et al.*, 2021; la Cour Freiesleben *et al.*, 2021). Cosma *et al.* compared 100 cases of early miscarriage with 125 cases of continuous pregnancy and found no significant difference in the cumulative incidence rate of COVID-19 infection between the two groups. Logistic regression analysis also indicated that COVID-19 infection was not an independent risk factor for early pregnancy loss (Cosma *et al.*, 2021). A Danish study of only 18 cases of early pregnancy in women infected with COVID-19 did not observe an increase in the miscarriage rate (la Cour Freiesleben *et al.*, 2021). Similarly, a large-sample population study conducted in 2022, after matching by factors of age and pregnancy season, found that COVID-19 infection in early pregnancy did not increase the risk of miscarriage (Calvert *et al.*, 2022). On the contrary, a study from the United Kingdom, which relied on patient self-reported diagnosis of SARS-CoV-2 and whether or not they had a miscarriage before 13 weeks of pregnancy, suggested that maternal infection with SARS-CoV-2 in the first trimester was associated with an increased risk of early miscarriage (Balachandren *et al.*, 2022).

The literature mentioned above primarily focuses on women with natural conceptions, but there is a lack of research on the impact of COVID-19 infection on the miscarriage during early pregnancy after IVF cycles. In the field of IVF research, most studies have compared IVF outcomes between patients before and after the COVID-19 pandemic, or between those with a history of COVID-19 infection and those without (Wang *et al.*, 2021; Setti *et al.*, 2021). Limited data are thus available on the specific effect of COVID-19 infection on miscarriage rates in IVF-conceived pregnancies. It is important to note that patients undergoing IVF treatment have a higher miscarriage rate compared to women with naturally conceived pregnancies (Wang *et al.*, 2004). This might be attributed to the underlying causes of infertility such as endometriosis and polycystic ovary syndrome (PCOS) that themselves increase the risk of miscarriage (Luo *et al.*, 2017; Stanekova *et al.*, 2018). Therefore, it remains unclear whether COVID-19 infection significantly increases the miscarriage rate in these patients. To our knowledge, only a small-sample study including 30 cases showed that the miscarriage rate was not affected after COVID-19 infection after IVF (Kabalkin *et al.*, 2022). This further emphasizes the need for additional research to shed light on this topic.

After implementing the ‘zero-COVID’ policy for more than two years, China changed its response strategy for COVID-19 on 7 December 2022, by announcing ‘10 new measures’, aiming to minimize the impact of the pandemic on economic and social development. The new policy included home isolation or quarantine for individuals infected with COVID-19 with mild symptoms or those who were asymptomatic and the termination of region-wide mass testing. Consequently, there was a rapid outbreak of SARS-CoV-2 infection spread across the country, with ∼70% of the population getting infected within three weeks (Liang *et al.*, 2023). Although we have recently discovered that viral RNA was not detected in plasma, villus, or fetal samples after COVID-19 infection at 0–8 gestational weeks (Xu *et al.*, 2023), it is important to note that this does not completely rule out the possibility of an increased risk of miscarriage, since previous studies have suggested that changes in the maternal immune system and cytokine storm caused by COVID-19 infection may potentially impact fetal development and lead to adverse outcomes (Cavalcante *et al.*, 2021).

To this aim, we conducted a strictly matched case–control study to further explore whether COVID-19 infection in IVF-conceived early pregnancy is associated with an increased risk of miscarriage.

## Materials and methods

### Study design and participants

We performed a matched cohort study of pregnant women who had undergone embryo transfer in Guangzhou Women and Children’s Hospital, a public assisted reproductive technology center in China. This matched cohort study was approved by the Independent Ethics Committee of Guangzhou Women and Children’s Hospital.

Approximately 1 month following the introduction of the new policy in China, a telephone questionnaire survey was conducted with all potential clinically pregnant women after embryo transfer in our center from 6 to 11 January 2023. Information about the characteristics of the COVID-19 disease (confirmed by antigen or polymerase chain reaction test), pregnancy viability, and pregnancy complications were recorded (Supplementary File S1). Due to the short interval between the follow-up and the outbreak of the epidemic, as well as the attention paid by the Chinese government and the masses to the epidemic, all patients could clearly recall the incidence of the disease. The date of onset of COVID-19 infection was defined as the date the woman’s first positive sample was taken. Patients who contracted COVID-19 before 20 weeks gestation were included for preliminary analysis. Women who met any of the following criteria were excluded from this study: (i) suspected SARS-CoV-2 infection lacking confirmation of nucleic acid or antigen testing; (ii) miscarriage diagnosed prior to the onset of infection; (iii) preimplantation genetic testing (PGT) cycles; (iv) confirmed ectopic pregnancy cases; and (v) cases of termination of pregnancy for medical or other reasons.

Prior to the implementation of the new epidemic prevention policy, the Chinese government adopted strict epidemic prevention management and control policy; hence, IVF services in mainland China were carried out in a non-epidemic state from the beginning of 2020 to November 2022. Therefore, we matched the infected pregnant women to uninfected historically pregnant controls to avoid any misclassification of women with undiagnosed infections as uninfected. The control group was selected from individuals who underwent embryo transfer from 1 January 2018 to 31 May 2022; hence, all included pregnancies could be observed up to 24 weeks of gestation or the end of pregnancy if earlier, before the new epidemic policy.

The matched control group was selected by the following criteria: (i) age (±1 year); (ii) number of gestational sacs; (iii) number of previous miscarriages; (iv) BMI (±2 kg/cm^2^); (v) main causes of infertility (ovulatory disorder, endometriosis, tubal factor, male factor, mixed factors); (vi) gestational week (e.g. if a woman was infected at 9 weeks of gestation, she would be matched to historically control women with an ongoing pregnancy at 9 weeks gestation); (vii) fresh versus frozen embryo transfer; (viii) endometrial thickness; (ix) type of protocol used for ovarian stimulation in fresh embryo transfers or endometrial preparation in frozen embryo transfers; and (x) embryo condition (embryo number, stage, and quality). We required exact matching for criteria 1–7 and 3:1 matching was possible for all groups due to sufficient controls. For criteria 8–10, we attempted matching as closely as possible and in most cases were able to match two of these criteria. To reduce the introduction of potential bias, researchers were blinded to reproductive outcomes during the matching process. If multiple patients fitted the criteria, one was chosen at random. Nearly all (∼99.5%) of the local population of patients who received IVF treatment in our center were Han Chinese; hence, these ethnicity data were not collected in the present study.

### Follow-up after embryo transfer

The level of serum β-hCG was measured at 12–14 days after embryo transfer. If the pregnancy test was positive (serum β-hCG >50 mIU/ml), transvaginal ultrasound was performed 2 weeks later to determine fetal viability and the number of gestational sacs. A transvaginal ultrasound was performed after another 2 weeks later to determine the fetal viability, if the first transvaginal ultrasound was normal. A nuchal translucency scan and fetal malformations screening were scheduled at gestational weeks 11–13 and (approximately) 22–24, respectively. All the IVF cycles are required to achieve a 100% follow-up rate after embryo transfer in our center.

### Outcome assessments

A clinical pregnancy was defined as the presence of an intrauterine gestational sac (with or without a fetal heartbeat) on ultrasonography during the first trimester. A clinical miscarriage was defined as the loss of a clinical pregnancy that takes place between the detection of the gestational sac and before 24 weeks’ gestational age. The last follow-up period for the infection group was 10–15 June 2023, ensuring that all pregnant women were at >24 weeks gestation.

### Statistical analysis

The 95% CI of miscarriage rate was estimated using the Clopper–Pearson exact method. The miscarriage rate between two groups was compared using the Fisher’s exact method. A *P*-value of <0.05 was considered statistically significant. To explore the impact of gestational age at the onset of SARS-CoV-2 infection on miscarriage, women in the infection group were stratified into three subgroups: 0–7 weeks 6 days gestation, 8–11 weeks 6 days gestation, and 12–19 weeks 6 days gestation.

## Results

**Figure 1.**
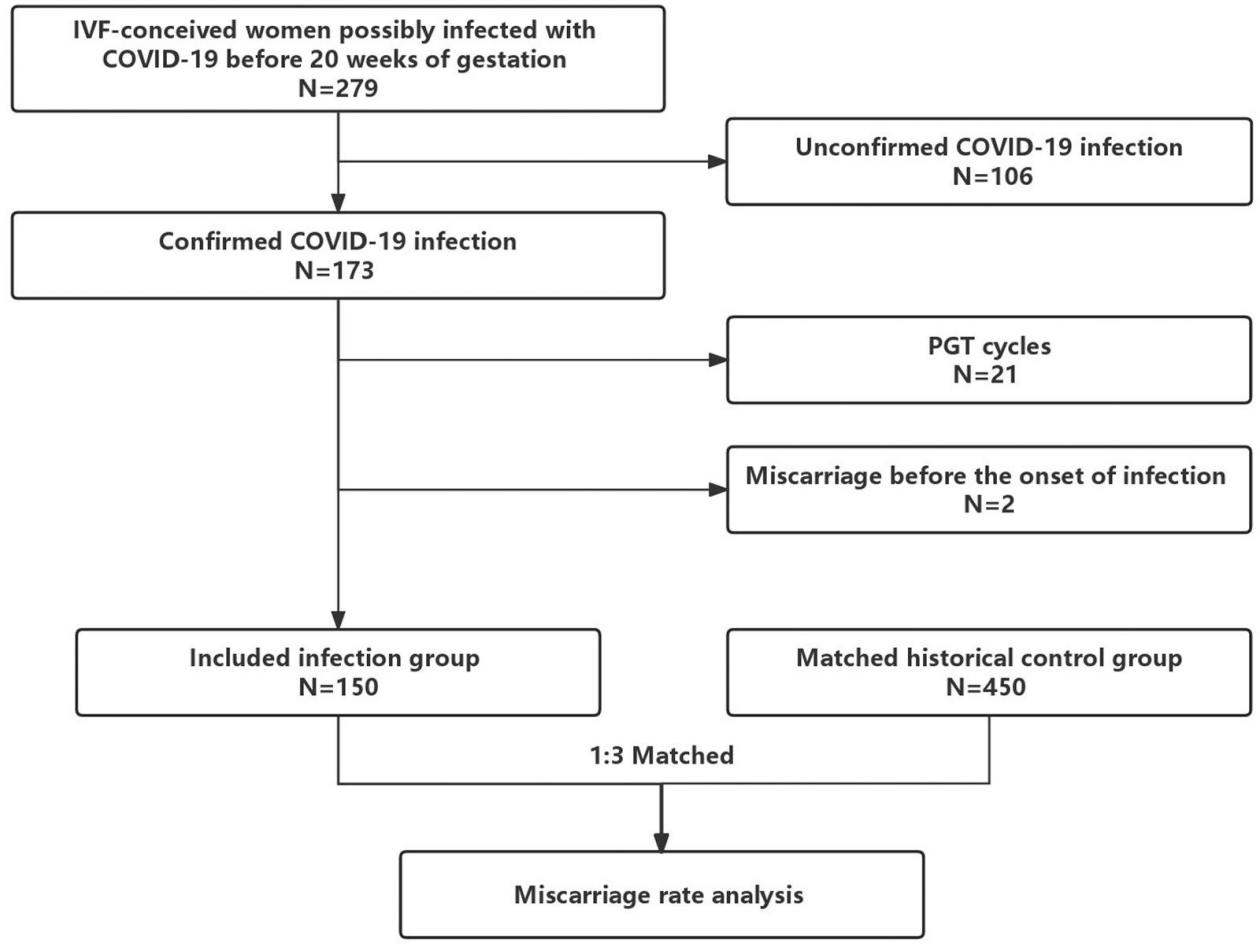
**Flow chart of participant screening and matching**. COVID-19: coronavirus disease 2019; PGT: preimplantation genetic testing.

A total of 279 women who had conceived by IVF were within their first 20 weeks gestation from 6 December 2022 to 10 January 2023; of these, 173 women were diagnosed with a confirmed COVID-19 infection. There were 150 pregnant women finally included in the infection group (Fig. 1), deriving from 32 fresh embryo transfer cycles and 118 frozen embryo transfer cycles. All these women reported only mild COVID-19 symptoms during their active infection period. The systemic symptoms improved significantly within 1 week in all of these patients, and none of them were hospitalized due to COVID-19 infection.

The study also included 450 historically pregnant controls matched to the infection group at a ratio of 3:1. The cycle characteristics of the infection group and the matched historical controls are presented in Table 1.

**Table 1.** Baseline characteristics.

Item	Infection group	Matched control group
	(n = 150)	(n = 450)
Age (years)	31.5 ± 3.3	31.5 ± 3.3
No. of previous miscarriages		
0	119	357
1	30	90
2	1	3
No. of gestational sacs		
1	127	381
2	23	69
BMI (kg/m^2^)	21.2 ± 2.8	21.1 ± 2.5
Cause of infertility		
Ovulatory disorder	40 (26.7%)	120 (26.7%)
Endometriosis	25 (16.7%)	75 (16.7%)
Tubal factors	36 (24.0%)	108 (24.0%)
Male	24 (16.0%)	72 (16.0%)
Mixed factors	25(16.7%)	75 (16.7%)
Endometrium thickness (mm)	10.3 ± 2.3	10.3 ± 2.4
Cycle type		
Cycles of fresh embryos transfer	32 (21.3%)	96 (21.3%)
Cycles of frozen embryos transfer	118 (78.7%)	354 (78.7%)

No significant difference was observed in the overall incidence of miscarriage between the infection group and the matched control group (4.7% versus 5.8%, *P* = 0.68) (Table 2). Similarly, we found no significant difference in the incidence of miscarriage between the infection group and the matched control group in the three subgroups (9.8% versus 13.8%, *P* = 0.60; 5.4% versus 4.5%, *P* = 1.00 and 1.4% versus 1.9%, *P* = 1.00, respectively) (Table 2).

**Table 2.** Gestational age at the onset of SARS-CoV-2 infection and miscarriage rate.

Item	Miscarriage rate		

Gestational week at the onset of infection	Infection group	Matched control group	*P*
	(n = 150)	(n = 450)	
0–19 + 6	4.7% (7/150)	5.8% (26/450)	0.68
	95% CI 1.9–9.4	95% CI 3.8–8.4%	
0–7 + 6	9.8% (4/41)	13.8% (17/123)	0.60
	95% CI 2.7–23.1%	95% CI 8.3–21.2%	
8–11 + 6	5.4% (2/37)	4.5% (5/111)	1.00
	95% CI 0.7–8.2%	95% CI 1.5–10.2%	
12–19 + 6	1.4% (1/72)	1.9% (4/216)	1.00
	95% CI 0–7.5%	95% CI 0.5–4.7%	

## Discussion

To the best of our knowledge, this is the first report, using a strictly matched case–control method, to investigate the association between SARS-CoV-2 infections in early pregnancy and miscarriage in IVF cycles. Our study found no evidence of an increased risk for miscarriage after COVID-19 infection in early pregnancy.

Previous studies have demonstrated that there are many confounding factors when analyzing the miscarriage rate, such as female age (Magnus *et al.*, 2019), BMI (Cozzolino *et al.*, 2021), previous miscarriages (Coomarasamy *et al.*, 2020), gestational age (Arck *et al.*, 2008), number of gestational sacs (especially in IVF achieved pregnancy) (Tummers *et al.*, 2003), and gynecological disease, such as endometriosis and PCOS (Luo *et al.*, 2017; Stanekova *et al.*, 2018). Therefore, these factors were strictly matched in our study to ensure a balanced representation between the infection and control groups, which ultimately enhanced the reliability of our results.

After strict matching, we found that there was no significant difference in the miscarriage rate between the two groups, regardless of the infection of COVID-19 in any gestational week. Consistent with our results, Calvert *et al.* (2022) also found that COVID-19 infection in early pregnancy did not increase the risk of miscarriage. However, their research had limitations in effectively controlling for confounding factors. First, they only conducted matching based on maternal age and gestational age at infection, whereas nearly all the confounding factors were matched in our study. Second, their analysis focused on naturally conceiving individuals, which often results in data loss during the very early stages of pregnancy, potentially introducing data bias. In contrast, our study included only pregnancies achieved through IVF, with a complete follow-up, enabling us to identify all early pregnancy losses accurately. Third, it is essential to note that the assessment of gestational age in naturally pregnant women is prone to inaccuracy. Furthermore, due to inadequate follow-up, determining the precise gestational age at the time of miscarriage becomes challenging, thus potentially compromising the assessment of the relationship between infection and miscarriage. Conversely, in our study, we could accurately determine the gestational age of patients based on the timing of embryo transfer. Moreover, in our center, we conducted ultrasound examinations at ∼6, 8, 12, and 24 weeks of pregnancy, which significantly minimized misjudgments regarding gestational age at pregnancy loss. Our strict follow-up procedures greatly enhanced the accuracy of the data and ultimately increased the reliability of our results.

On the contrary, a study conducted in the UK involved women who conceived during the COVID-19 pandemic and relied on self-reported information regarding SARS-CoV-2 diagnosis and miscarriage within the first 13 weeks of pregnancy. This study reported an increased risk of miscarriage, but the findings were accompanied with wide confidence intervals and were susceptible to recall bias (adjusted risk ratio = 1.7, 95% CI = 1.0–3.0) (Balachandren *et al.*, 2022).

A recent study showed that COVID-19 infection resulted in decreased embryo and blastocyst quality during IVF treatment (Tian *et al.*, 2023). However, patients included in our study were those who were infected only after embryo transfer and subsequently achieving a clinical pregnancy. In this case, when analyzing the relationship between infection and miscarriage, we mainly focus on the maternal impact of the infection. Notably, our recent research discovered that viral RNA was not detected in any plasma, villus, and fetal samples after COVID-19 infection at 0–8 gestational weeks (Xu *et al.*, 2023). Therefore, our previous result also supports the current conclusion.

Spontaneous miscarriage affects between 11% and 20% of pregnancies (Ammon Avalos *et al.*, 2012). However, our study observed considerably lower miscarriage rates, at ∼5%, in both patient groups. The main reason is that the risk of pregnancy loss decreases with advancing gestational age (Arck *et al.*, 2008), and nearly half of the patients we included were those with gestational age exceeding 12 weeks. Moreover, the individuals we included in our study were of comparatively younger ages, reasonable BMI, and the majority of them lacked a history of miscarriage. These elements collectively contributed to the notably decreased miscarriage rate observed in our study.

The main strength of the present study is the effective research method, with nearly all the confounding factors being strictly matched. In addition, for patients who achieved pregnancy through IVF, we can accurately calculate their gestational age based on the time of embryo transfer. Our center conducts complete follow-up on patients after embryo transfer, and ultrasound examination is performed at ∼6, 8, and 12 weeks of pregnancy to determine the gestational stage of pregnancy loss for all pregnant women. Moreover, the epidemic outbreak occurred just after the change in epidemic prevention and control policies in China. We tracked patients one month after the outbreak, allowing them to recall the timing of their infection with clarity. This enabled us to accurately determine the gestational age at the time of infection, which is crucial for our analysis.

There are some limitations that need to be considered. First, the sample size is not large enough, hence caution should be taken when attempting to draw any definitive conclusions. Nonetheless, as far as we know, this is the largest sample study of COVID-19 infection in early pregnancy after IVF on miscarriage rate. Second, multiple pregnancies were included in our study, but chronicity was not analyzed, so we could not rule out possible impact of multiple pregnancies on miscarriage rates. Third, the timeframe selected for the control group is different from that of the infection group. Although there were no differences in the IVF procedures, including ovarian stimulation protocols, culture medium used, embryo freezing and thawing methods, etc., we cannot completely exclude the impact of the different timeframe on our results.

## Conclusions

Our study found no evidence of an increased risk for miscarriage after COVID-19 infection in early pregnancy. However, caution should be taken due to the small sample size. Our findings may represent a guide for reproductive physicians and obstetricians during preconception and early pregnancy counseling. Additionally, our results provide meaningful clinical evidence that COVID-19 infection may not be a causative factor for miscarriage in women who have experienced both COVID-19 infection and miscarriage during early pregnancy. This information can offer reassurance and support to those individuals who may have concerns about the relationship between COVID-19 and miscarriage.

## Supplementary Material

hoae024_Supplementary_Data

## Data Availability

The data that support the findings are available from the corresponding author, upon reasonable request.
