# Supplementary material for: SARS-CoV-2 infection in IVF-conceived early pregnancy and the risk of miscarriage: a matched retrospective cohort study
Source: Hum Reprod Open. 2024 Apr 23;2024(2):hoae024. doi: 10.1093/hropen/hoae024 (PMC11099652; doi:10.1093/hropen/hoae024)
Supplement: hoae024_Supplementary_Data [file hoae024_supplementary_data.docx]

**Questionnaire on COVID-19 and IVF**

Patient Name________; Spouse Name___________; IVF system number_______

Questionnaire date: ___________; questionnaire number ______________

In order to investigate the impact of COVID-19 on IVF-conceived early pregnancy, we kindly ask you to complete this questionnaire. The information provided will be kept strictly confidential. Thank you for your cooperation!

**1. whether infected with COVID-19**

1. Yes

b. No

**2. Diagnosis method and date:**

① Diagnosis method :

1. Nucleic acid test
2. Antigen test

② Date of Diagnosis:

Nucleic acid: ________________________; Antigen: ____________________

**3. Symptoms during COVID-19 infection**

1. Fever, maximum ____ ℃, start date: ___________________
2. cough
3. shortness of breath
4. fatigue
5. stuffy nose
6. headache
7. Gastrointestinal symptoms (nausea, vomiting, diarrhea)
8. chest pain
9. muscle ache
10. eye pain
11. sore throat
12. Brain symptoms (dizziness, hallucinations, difficulty concentrating)
13. shivering (no fever)
14. flu-like feeling
15. Other symptoms: _________________________

**4. How serious do you feel about the symptoms of COVID-19 infection?**

a. light; b. medium;c. heavy; d. no feeling

**5. Treatment and recovery**

1. Whether hospital visit?
2. yes b. no
3. Drug name, dose, and frequency: ____________________________________________________

③ In which day when you start to feel that your general symptoms have improved significantly:

a. Day 1 b. Day 2 c. Day 3 d. Day 4 e. Day 5 f. More than 5 days

④ Date of COVID-19 detection turned negative: ________________________________

Diagnosis method: a. Nucleic acid test; b. Antigen test

____________________
